# Insights into skeletal stem cells

**DOI:** 10.1038/s41413-022-00235-8

**Published:** 2022-10-19

**Authors:** Qiwen Li, Ruoshi Xu, Kexin Lei, Quan Yuan

**Affiliations:** State Key Laboratory of Oral Diseases, National Clinical Research Center for Oral Diseases, West China Hospital of Stomatology, Sichuan University, Chengdu, 610041 China

**Keywords:** Bone, Physiology

## Abstract

The tissue-resident skeletal stem cells (SSCs), which are self-renewal and multipotent, continuously provide cells (including chondrocytes, bone cells, marrow adipocytes, and stromal cells) for the development and homeostasis of the skeletal system. In recent decade, utilizing fluorescence-activated cell sorting, lineage tracing, and single-cell sequencing, studies have identified various types of SSCs, plotted the lineage commitment trajectory, and partially revealed their properties under physiological and pathological conditions. In this review, we retrospect to SSCs identification and functional studies. We discuss the principles and approaches to identify bona fide SSCs, highlighting pioneering findings that plot the lineage atlas of SSCs. The roles of SSCs and progenitors in long bone, craniofacial tissues, and periosteum are systematically discussed. We further focus on disputes and challenges in SSC research.

## Introduction

The skeleton is comprised of mineralized matrix that protects organ and facilitates body movement. It is a reservoir for calcium and phosphate, regulating the systematic mineral ion metabolism.^1,2^ The bone marrow harbors niches for hematopoiesis.^3^ Bone also regulates systemic metabolism and closely interacts with other organs such as brain, kidney, intestine, and liver.^4–10^ Over the decades, the skeletal stem cells (SSCs) have gained great attention based on the recognition that SSCs are situated at the apex of the lineage differentiation trajectory and continuously provide cells for bone development, homeostasis, and injury repair.^11–14^ Understanding the properties and lineage commitment of SSCs help revealing the nature of organogenesis and aiding in the treatment of disorders such as congenital anomalies (e.g., dwarfism, cleidocranial dysplasia), age-related diseases (e.g., osteoporosis, osteoarthritis), tumor (e.g., chondromas, osteochondromas) and rare genetic diseases (e.g., fibrodysplasia ossificans progressiva, progressive osseous heteroplasia).^15–23^ However, the search for bona fide stem cells has been a long-striving goal in the fields of bone research. The intricate developmental phases, heterogenous cell types, and mineralized structure of the skeletal system make this work extremely challenging.^24,25^

### Definition and characterization

The concept of SSCs or mesenchymal stem cells (MSCs) originates from the groundbreaking discovery that heterotopic transplantation of total bone marrow cell suspensions or boneless marrow fragments formed ossicles and reconstructed hematopoietic and reticular structures.^26,27^ This capacity was later ascribed to a population of self-renewable and multipotent stromal cells, which were defined as “mesenchymal stem cells”.^28–30^ The International Society of Cellular Therapies has proposed a minimal criteria to define MSCs, wherein the cells with plastic-adherent and tri-lineage differentiation ability (osteoblasts, chondrocytes, adipocytes), as well as with the expression of subsets of immunophenotypic makers (CD73, CD90, and CD105, but not CD34, CD45, CD14/CD11b, CD79a/CD19, and HLA-DR) were regarded as putative MSCs.^31^ With time, the MSC concept was extended to other tissues, such as fat, muscle, and dental pulp.^32–34^ It seems that researchers could always culture out a plate of “stem cells” from any mesenchymal tissue.^35^ However, this definition and characterization of MSCs was later considered inadequate, as it indeed yielded highly a heterogenous and unidentified population.^11,12^ Ectopic transplantation of these “stem cells” often failed to form a chondro-osteo structure or support a hematopoietic environment in vivo.^12^ The ambiguity of MSCs further jeopardizes the clinical translation as stem cell therapy failed to reach a predictable outcome.^36^

To address these issues, Paolo Bianco proposed the term “skeletal stem cells” to indicate the skeletal tissues-resident, self-renewable and multipotent cells that generate cartilage, bone, hematopoiesis-supporting stroma and marrow adipocytes.^12,37^ Importantly, it was advocated that, along with colony formation units (CFUs) and tri-lineage differentiation test in vitro, the self-renewal and multipotent properties should be tested with rigorous in vivo assays. Serial transplantation study is a golden standard to verify bona fide SSCs.^11^ Cells are sorted with presumptive stem cell surface markers and transplanted ectopically, and are then evaluated for the formation of the bone and bone marrow components. Presumptive SSCs again are sorted from the newly formed bone and then secondarily transplanted, to test the ability to form complete skeletal components.^11,38–40^ Likewise, the multipotency of presumptive SSCs are analyzed in situ, by co-labeling with different lineage markers. EdU (5-ethynyl-2′-deoxyuridine)/BrdU (5-bromo-2′-deoxyuridine) retention test further helps to verify the slow-cycling cells, generally regarded as stem cells.^41,42^ Overall, SSCs and MSCs indicate different cell populations and their meanings are not interchangeable. To some extent, SSCs can be regarded as more homogeneous subpopulations of MSCs. The use of “SSCs” is recommended to define the self-renewable and multipotent cells that are restricted to skeletal system and display explicit surface markers.

### Identification and isolation

Two approaches are now widely used to identify SSCs, fluorescence-activated cell sorting (FACS) and transplantation assay, and *Cre-loxP* system-driven lineage tracing strategy.^43,44^ The identification and isolation of different lineages by FACS and transplantation assay is routinely used in hematopoietic domain, which facilitates the classification of differentiation trajectory and precise elucidation of homogenous cells subsets without contamination.^45^ However, as stem cells and progenitors are differentially labeled, often with several markers, visualization and functional study in situ are much limited. Besides, it is reported that in native hematopoietic environment, the maintenance of blood production during adulthood is contributed mainly by long-lived lineage-restricted progenitors rather than hematopoietic stem cells which are classically defined by transplantation assay into lethally irradiated mice.^46^

Lineage tracing, on the other hand, enables the spatial and temporal observation of SSCs and progenies in an unperturbed manner.^43^ Diverse site-specific recombinases are developed for lineage tracing, such as *Cre-loxP*, *Dre-rox*, and *Flp-frt.*^47–50^ *Cre-loxP* technology-based mouse models are most frequently constructed in the bone field. Briefly, Cre recombinase expressed under the control of different transgenes removes the *loxP* site and mediates the expression of reporter genes.^50^ Using the tamoxifen-induced *CreER* expression system, we are able to study the stem cells in a restricted time window.^51^ Besides, reporters directly driven by promoters of transgenes are widely used in combination with *Cre-loxP* technology, such as Col1(2.3 kb)-GFP.^52^ *Dre-rox* system is compatible with *Cre-loxP* system, and orthogonal recombinase system is developed that facilitates the label of intersect or subtractive parts of cell populations.^53,54^

Different reporter mice are available, such as *R26R*-LacZ (β-galactosidase), *R26R*-EYFP (yellow fluorescent protein), *R26R*-tdTomato, double-fluorescent mT/mG, and four fluorescent R26R-Confetti/Brainbow2.1 (Table 1).^55–59^ Especially, the mT/mG mice express membrane-targeted tandem dimer Tomato (mT) and membrane-targeted green fluorescent protein (mG) before and after Cre-mediated excision respectively.^57^ The *R26R*-Confetti mice stochastically express one of the YFP, tdTomato, GFP, and CFP (cyan fluorescent protein) after Cre-mediated excision.^58^ A single SSC and its progeny express the same fluorescent protein, so we can evaluate the clonogenic ability using *R26R*-Confetti mice.^58^ Nonetheless, the non-specificity of transgenes is an issue in lineage tracing.^48^ Occasionally, unwanted cell types are simultaneously labeled.^60^ Poor recombination is observed in some *Cre*/*CreER* mice as well.^61^ *Osterix* (*Osx*)*-Cre* mouse, a widely used transgenic model, unexpectedly displayed bone developmental defects.^62^ Besides, cells labeled by site-specific recombinase include SSCs and descendants, so it is almost impossible to sort out a homogenous stem cell population with a single transgene. Therefore, a thorough characterization of SSCs relies on both cell sorting and lineage tracing.

**Table 1:** Comparison of different reporter mice based on *Cre/loxP* system

Reporter mice	Visualization	Features	References
R26R-LacZ	X-gal staining	Capable of whole-mount visualization; staining cannot be performed in live-tissue	^55^
*R26R*-EYFP *R26R*-tdTomato	Fluorescence	Capable of live-tissue visualization and co-staining with other markers	^56,58^
Double-fluorescent mT/mG	Fluorescence	Cells express tdTomato (mT) and GFP (mG) before and after Cre-mediated excision respectively; the non-recombined cells function as an internal control; suitable for mosaic analysis	^57^
*R26R*-Confetti/Brainbow2.1	Fluorescence; confocal microscope recommended	Usually bred with *CreER* and the recombination efficiency is affected by tamoxifen; stochastic recombination; capable of multicolor visualization (green, yellow, red, blue), distinguishment of adjacent cells, and clonogenic assay	^58,63^
*Transgenes*-GFP/mCherry/DsRed	Fluorescence	Expression is directly driven by transgenes and is *Cre/loxP* system-independent, widely used in combination with *Cre/loxP* system	^41,59,64^

### Current understanding in SSC research

Several goals are to be achieved in SSC research. The first is to plot the entire differentiation trajectory of SSCs based on authentic surface markers. The second is to answer how dysfunction of SSCs leads to pathogenesis. The third is to explore major factors that determine the fate commitment of SSCs.

Several studies have rigorously identified and tested the bona fide SSCs using either a combination of immunophenotypic markers or a specific promotor/enhancer that drives transgene expression.^38,39,41,64–70^ The lineage differentiation trajectory of several types of SSCs have been plotted, though their overlap and difference require clarification.^38,66,71–74^ The opinion that a single SSC lineage gives rise to all bone components is an oversimplification. Indeed, accumulating evidence indicates that more than one types of SSCs contribute to skeletal development and homeostasis at different stages (we will discuss about it in “Disputes and challenges of SSCs”). For example, during embryonic development, different waves of progenitors emerge to support rapid bone growth. The *type II collagen* (*Col2*)-*Cre*-labeled perichondrial and growth plate progenitors and *Osx-Cre*-labeled progenitors in periosteum and primary spongiosa actively participate in bone growth and bone marrow formation.^67,69,75,76^ With growth, these early progenitors are replaced by long-lived SSCs such as leptin-receptor-expressing (LepR^+^) perivascular cells.^64,77^

Studies have revealed several postnatal stem cell niches that support SSCs self-renewal, including the perivascular niches, growth plate niches, and periosteal niches.^42,60,71,78^ Despite divergent microenvironment that regulates SSCs behavior, SSCs spontaneously differentiate into osteoblasts.^79^ In fact, the intrinsic ability of SSCs to form bone rather than adipocytes is established epigenetically, in which the chromatin accessibility is predetermined for osteogenesis. The adipogenic process instead requires a substantial remodeling of the chromatin landscape where enhancers are activated de novo.^79^ It is currently unclear why SSCs possess different ability to generate chondrocytes, adipocytes and stromal cells. One possible explanation is the niches. For example, growth plate SSCs sequentially differentiate into chondrocytes and then osteoblasts, but perisinusoidal SSCs differentiate into either osteoblasts or adipocytes in a mutually exclusive way.^41,80^ The cells at growth plate niches are predominantly regulated by factors like fibroblast growth factors (FGF), Wnt, parathyroid hormone-related protein (PTHrP), and Indian hedgehog (Ihh), whereas SSCs residing in the bone marrow niches actively interact with endothelial and hematopoietic cells, and therefore possess a different transcriptional landscape and differentiation trajectory.^73,81–86^

## Skeletal stem cells in long bones

### Markers of SSCs

Various surface markers for SSCs and lineage-restricted progenitors have been identified. Here we summarize these key findings of both human and mice based on FACS (Tables 2 and 3) and lineage tracing (Table 4). Initially identified in human bone marrow and dental pulp, STRO-1^+^ perivascular stem cells were positive for α-smooth muscle actin (αSMA) and CD146.^87^ The pioneering study to identify a functional human SSC group to support hematopoietic microenvironment is by Bianco Laboratory.^39^ They identified a group of subendothelial/adventitial reticular CD45^−^CD146^+^ SSCs with in vivo self-renewal and clonogenic properties. All CFU-fibroblasts (CFU-Fs) selected from unfractionated bone marrow showed high level of CD146 expression. CD45^−^CD146^+^ SSCs transplanted subcutaneously repetitively gave rise to bone and hematopoiesis-associated stromal cells. This study also provides a standard template to functionally characterize SSCs in vitro and in vivo.^39^

**Table 2:** Human skeletal stem cells labeled by immunophenotypic markers

Surface markers	Location	Features	References
STRO-1^+^	Perivascular	STRO-1^+^, CD146^+^, α-SMA^+^	^87^
CD146^+^	Perisinusoidal, subendothelial/adventitial	Bone formation, hematopoiesis-supportive	^78^
PDGFRα^+^CD51^+^	perivascular	NESTIN^+^, CD146^+^, bone formation, hematopoiesis-supportive	^88^
PDPN^+^CD146^−^CD73^+^CD164^+^	Fetal growth plate; adipose stroma and iPSCs when induced with BMP2	CD45^−^, CD235a^−^, Tie2^−^, CD31^−^; bone, cartilage and stroma formation (but not adipose tissue); hematopoiesis-supportive; fracture repair	^66^
PDGFRA^low/−^PDPN^+^CADM1^+^	Fetal perichondrium, bone marrow	CD45^−^, CD31^−^, CD235a^−^; bone and cartilage formation; do not support hematopoiesis; identified in mouse long bones at E15.5 as well	^90^

**Table 3:** Mouse skeletal stem cells labeled by immunophenotypic markers

Surface markers	Location	Features	References
PDGFRα^+^Sca1^+^	Perivascular	Bone and adipose formation, hematopoiesis-supportive	^65^
CD45^−^Ter119^−^Tie2^−^AlphaV^+^Thy^−^6C3^−^CD105^−^CD200^+^	Femoral growth plate (mSSCs can be isolated from ribs and sternum as well)	Bone, cartilage, and stroma formation (but not adipose tissue); hematopoiesis-supportive; fracture repair	^38^
CD45^−^CD31^−^Sca1^+^CD24^+^	Perivascular	PDGFRα^+^; bone, cartilage, and adipocytes formation; hematopoiesis-supportive	^72^
CD45^−^Ter119^−^CD31^−^CD166^−^CD146^−^Sca1^+^	Mainly on the endosteal surface of trabecular bone in epiphysis	Bone and stroma formation, hematopoiesis-supportive	^74^

**Table 4:** Skeletal stem cells and progenitors labeled by transgenes

Transgenes	Location	Features	References
*Prx1-Cre*	Throughout limb bud mesenchyme, a subset of craniofacial mesenchyme	*Prx1-Cre*-expressing cells are enriched for all CFU-Fs in bone marrow	^64,107^
*Sox9-Cre*	Limb bud mesenchyme	*Sox9-Cre*-expressing cells generate cartilage, bone, tendon and synovium	^108^
*Col2-Cre* *Col2-Cre*^*ER*^ *Acan-Cre*^*ER*^ *Sox9-Cre*^*ER*^	Perichondrium, growth cartilage	*Col2-Cre*-expressing cells generate cartilage, bone, Cxcl12-abundant stromal cells, and adipocytes. Early postnatal cells marked by *Col2-Cre*^*ER*^, *Acan-Cre*^*ER*^ and *Sox9-Cre*^*ER*^ generate long-term progenitors in bone marrow.	^67,68^
*Gli1-Cre*^*ERT2*^	E14.5: perichondrium 1 month: articular cartilage, upper layers of growth plate, perichondrium and chondro-osseous junction	Postnatal metaphyseal Gli1^+^ cells express CD146/Mcam, CD44, CD106/Vcam1, Pdgfrα, and Lepr; generate bone, stroma and adipocytes	^98^
*Hoxa11-Cre*^*ERT2*^ *Hoxa11*-EGFP	The outer periosteum and bone marrow of zeugopod	Hoxa11-expressing cells generate postnatal SSCs marked by *LepR-Cre* and *Osx-Cre*^*ER*^, and are PDGFRα^+^ and CD51^+^	^120,121^
*Nestin*-GFP	Perichondrial and perivascular in developing bone; perivascular at adult stage	*Nes*-GFP marks both nonendothelial and endothelial cells, and the nonendothelial Nes^+^ cells are osteoblastic in developing bone. Note that *Nes-Cre*/*Nes-Cre*^*ER*^ preferentially targets endothelial cells. Postnatal *Nes*-GFP^+^ mesenspheres are enriched for CFU-Fs; hematopoiesis-supportive.	^68,78^
*Osterix-Cre* *Osterix-Cre*^*ER*^	Perichondrium, growth cartilage	Osterix marks three waves of progenitors: 1. Fetal Osterix^+^ cells generate bone and transient stromal cells; 2. Perinatal Osterix^+^ cells generate bone and long-lived stromal cells (Cxcl12^+^, *Nes*-GFP^+^); 3. Adult Osterix^+^ cells contribute to osteo-lineage only.	^67,69^
*PTHrP-mCherry* *PTHrP-Cre*^*ER*^	Fetal stage: perichondrium Postnatal: resting zone of the growth plate	PTHrP-mCherry^+^ cells contain a large portion of CD45^−^Ter-119^−^Tie2^−^AlphaV^+^Thy^−^6C3^−^CD105^−^CD200^+^ mSSCs; PTHrP-expressing cells generate chondrocytes, osteoblasts and Cxcl12^+^ stromal cells, but not adipocytes; long-term SSCs after secondary ossification center formation	^41^
*FoxA2-Cre*	Top compartment of the resting zone of the growth plate	FoxA2^+^ cells are long-term and highly clonogenic; mainly contribute to the maintenance of growth plate turnover and regeneration	^119^
*Col10a1-Cre* *Col10a1-Cre*^*ERT2*^	Growth plate hypertrophic chondrocytes	Col10a1-expressing chondrocytes undergo de-differentiation to generate long-lived SSCs	^122^
*Pdgfrα-*H2BGFP	Bone marrow	Pdgfrα^+^ cells are highly enriched for CFU-F, but *Pdgfrα-Cre*^*ER*^ recombines poorly in bone marrow.	^61,64^
*Pdgfrβ-Cre*^*ERT2*^	Metaphysis, bone marrow, periosteum, a small fraction of growth plate cartilage	Perinatal *Pdgfrβ*^*+*^ cells are restricted to metaphysis, and juvenile *Pdgfrβ*^*+*^ cells are located at metaphysis and bone marrow; *Pdgfrβ*^*+*^ cells generate osteoprogenitors, chondrocytes and adipocytes; *Pdgfrα*^*+*^*β*^*+*^ metaphyseal SSCs generate diaphyseal SSCs.	^137^
*Gremlin1-Cre*^*ERT*^	Primitive mesenchyme, primary spongiosa at P1, non-perivascular	Gremlin1-expressing cells give rise to osteoblasts, chondrocytes and reticular stromal cells, but not adipocytes	^97^
*Kit*^*MerCreMer*^	Fetal chondrocytes, pre-osteoblasts, stromal cells	Fetal C-KIT^+^ cells generate ~20% postnatal LepR^+^ cells; *Kit*^*MerCreMer*^ does not label postnatal SSCs	^118^
*Cxcl12-Cre*^*ER*^	Perisinusoidal	*Cxcl12-Cre*^*ER*^-expressing cells remain quiescent physiologically, and activate to form osteoblasts in response to injury	^123^
*LepR-Cre* *LepR-Cre*^*ER*^	Perivascular	LepR^+^ cells are derived from fetal Col2^+^ cells; PDGFRα^+^, Prx1^+^, *Scf*-GFP^+^, Cxcl12-DsRed^high^, *Nes*-GFP^low^; highly enriched for CFU-Fs; major source of bone and adipocytes in adult mice; hematopoiesis-supportive	^64,77^
*CTSK-*mGFP *Ctsk-Cre*	Long bone and calvarial periosteum (endosteal *CTSK-*mGFP cells are osteoclasts)	Periosteal CTSK-mGFP^+^ cells contain TER119^-^CD31^−^CD45^−^THY1.2^−^6C3^−^CD200^+^CD105^−^ mSSCs; bone formation via intramembranous ossification physiologically; re-establish endochondral bone formation ability in response to injury	^71^
Mx1^+^αSMA^+^ (*Mx1-Cre;R26-Tdt;αSMA*-GFP)	Long bone and calvarial periosteum	~80% of periosteal CD31^−^CD45^−^TER119^−^Mx1^+^αSMA^+^ cells are CD105^+^CD140a^+^ SSCs; highly express *Runx2*, *Cxcl12*, *LepR*; CCL5-medaited migration to injury site	^99^
*Mx1-Cre*	Bone marrow (*Mx1-Cre* labels hematopoietic cells as well)	*Mx1-Cre*-expressing cells label a fraction of CD105^+^CD140a^+^ SSCs; multipotent in vitro, but only give rise to osteoblasts in vivo	^127^
LepR^+^osteolectin^+^ *Oln*-mTomato *Oln*^*iCreER*^	Periarteriolar	Almost all Oln-mTomato^+^ cells are LepR^+^; rapidly dividing, short-lived osteogenic precursors	^128^
*Adipoq-Cre* *Adipoq-Cre*^*ER*^	Bone marrow	A subpopulation of LepR^+^ cells; adipocytes progenitors; hematopoiesis-supportive; at least a subset of *Adipoq-Cre*-expressing cells are bipotent	^133,134,136^

Further study identified a small fraction of CD146^+^ cells that were PDGFRα^+^CD51^+^ in the bone marrow, which exhibited even higher clonogenic capacity. These PDGFRα^+^CD51^+^ cells were mainly present in fetal bone marrow and robustly expressed *NESTIN* and hematopoietic stem cell (HSC) maintenance genes.^88^ Moreover, a group of lin^−^/CD45^−^/CD271^+^ SSCs were identified.^89^ lin^−^/CD45^−^/CD271^+^ cells gave rise to bone and hematopoietic stroma when transplanted, regardless of CD146 expression. Interestingly, lin^−^/CD45^−^/CD271^+^/CD146^+^ SSCs were perivascular, while lin^−^/CD45^−^/CD271^+^/CD146^−/low^ SSCs were endosteal.^89^

In 2018, Chan et al. plotted lineage differentiation trajectory of human SSCs. Combining single-cell RNA sequencing (scRNA-seq) with FACS, they identified CD45^−^CD235a^−^Tie2^−^CD31^−^PDPN^+^CD146^−^CD73^+^CD164^+^ cells as the bona fide human SSCs, which were able to form ectopic ossicles with hematopoiesis-supporting marrow stroma even after serial renal capsule transplantation.^66^ Interestingly, these SSCs can be isolated not only from human fetal and adult bone, but also from bone morphogenetic protein 2 (BMP2)-treated adipose stroma and induced pluripotent stem cells (iPSCs). Comparison between CD146^+^ SSCs and PDPN^+^CD146^−^CD73^+^CD164^+^ SSCs showed a higher colony-forming ability of the latter pupulation.^66^

For mouse skeletal stem cells, Matsuzaki et al. identified a group of nonhematopoietic PDGFRα^+^Sca1^+^ SSCs from adult mouse bone marrow.^65^ These cells resided in a perivascular space and were highly enriched with CFU-Fs. A single PDGFRα^+^Sca1^+^ cell was sufficient to give rise to mesenchymal and endothelial lineages. When freshly isolated and transplanted into lethally irradiated mice with HSCs, PDGFRα^+^Sca1^+^ SSCs were capable of self-renewal and differentiating into osteoblasts, adipocytes, and HSC-supporting stromal cells. However, cultured PDGFRα^+^Sca1^+^ cells failed to engraft into the recipient mice.^65^

Chan et al. performed clonal assay using *Actin-Cre*^*ERT*^ driven rainbow reporter mouse and identified growth plate as a clonal region.^38,91^ A majority of growth plate cells were CD45^−^Ter119^−^Tie2^−^AlphaV^+^. Further screening identified eight subpopulations of CD45^−^Ter119^−^Tie2^−^AlphaV^+^ cells that gave rise to distinct components of bone. CD45^−^Ter119^−^Tie2^−^AlphaV^+^Thy^−^6C3^−^CD105^−^CD200^+^ (mouse skeletal stem cells, mSSCs) subpopulation was capable of self-renewal and generated other seven subpopulations through sequence of stages when transplanted beneath the renal capsule.^38^ A population of CD45^−^Ter119^−^CD31^−^CD166^−^CD146^−^Sca1^+^ (Sca1^+^) SSCs were also identified.^74^ Sca1^+^ SSCs generated CD45^−^Ter119^−^CD31^−^CD166^−^CD146^+^ (CD146^+^) intermediate progenitors and CD45^−^Ter119^−^CD31^−^CD166^+^CD146^+^ (CD166^+^) mature osteoprogenitors. Sca1^+^ SSCs also gave rise to C-X-C motif chemokine ligand 12-expressing (Cxcl12^+^) stromal cells and homed to bone marrow to support hematopoiesis when intravenously infused into sub-lethally irradiated mice.^74^

**Fig. 1:**
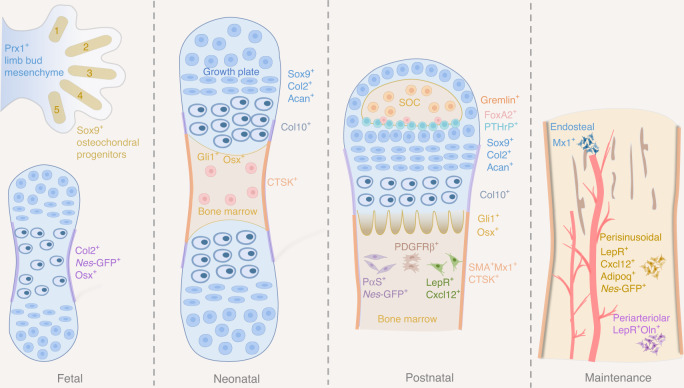
Schematics of skeletal stem cells and progenitors in mouse long bone. At fetal stage, limb bud mesenchymal cells express Prx1 and osteochondral progenitors are Sox9^+^. When fetal growth plate forms, Col2, *Nes*-GFP, and Osx mark progenitors at perichondrium. Neonatal long bone harbors various types of SSCs and progenitors, including Sox9^+^/Col2^+^/Acan^+^/Col10^+^ cells at growth plate, Gli1^+^/Osx^+^ cells at primary spongiosa, Ctsk^+^ cells at periosteum and Ranvier’s groove. Postnatal long bone is occupied with long-term SSCs and progenitors, including Grem1^+^/FoxA2^+^/PTHrP^+^/Sox9^+^/Col2^+^/Acan^+^/Col10^+^ cells at epiphysis and growth plate cartilage, Gli1^+^/Osx^+^ cells at primary spongiosa, PDGFRβ^+^/PαS^+^/*Nes*-GFP^+^/LepR^+^/Cxcl12^+^ cells in bone marrow, and αSMA^+^Mx1^+^/Ctsk^+^ cells at periosteum. Adult bone marrow is highly complex and contains endosteal, perisinusoidal, and periarteriolar niches, which harbor tissue-resident SSCs and progenitors essential for hematopoietic lineages

Utilizing *Cre-loxP* technology and fluorescent reporter mouse models, an array of SSCs and lineage-restricted progenitors are identified (Fig. 1). Here we briefly summarize the transgenes that specifically label these functional cells. Perivascular is common origin for mesenchymal stem cells, where several types of SSCs and progenitors were identified, including PDGFRα^+^, *Nestin*-GFP^+^ (*Nes*-GFP), LepR^+^, αSMA^+^, Ebf3^+^, and Cxcl12^+^ cells.^61,64,68,78,92–95^ *Nes-*GFP^+^ cells were perivascular and co-localized with HSCs, accounting for all CFU-Fs activity. The *Nes*-GFP^+^ “mesenspheres” could expand through serial heterotopic transplantation.^78,96^ Further study revealed some *Nes*-GFP^+^ cells were endothelial in embryonic perichondrium, and only the nonendothelial *Nes*-GFP^+^ cells generated osteoblast lineage. Meanwhile, cells targeted by *Nes-CreER* were predominantly endothelial in developing and adult bone marrow and the labeled cells make little contribution to early bone development, indicating the heterogeneity of Nes^+^ population.^68^ LepR^+^ SSCs mainly originated from fetal Col2^+^ precursors and gradually gave rise to bone cells and adipocytes in adult bone marrow.^64,77^

Growth plate and metaphyseal also harbor SSCs and progenitors, including Gremlin1^+^ (Grem1^+^), Sox9^+^, Aggrecan^+^ (Acan^+^), Col2^+^, FoxA2^+^, PTHrP^+^, Gli1^+^ and Osx^+^ cells.^41,67,69,97,98^ For instance, Grem1 marked a population of osteochondralreticular (OCR) stem cells in the metaphysis of long bone, which were more clonogenic than *Nes*-GFP^+^ SSCs.^97^ Embryonic perichondrium and postnatal periosteum are niches for SSCs.^67–69,71,99^ During early embryonic development, Sox9^+^, Col2^+^, *Nes*-GFP^+^, and Osx^+^ progenitors gave rise to bone marrow osteoblasts and stroma.^67–69^ Postnatal periosteum harbored cathepsin K-expressing (Ctsk^+^) SSCs and Mx1^+^αSMA^+^ SSCs which contributed to cortical bone formation and fracture repair (To be discussed in the session “Skeletal stem cells in periosteum”).^71,99^

### Emergence of SSCs in limb bud

Long bones are lateral plate mesoderm derived and the development starts with the emergence of limb buds at embryonic day (E) 9.5 (forelimb) or E10.5 (hindlimb).^25^ Morphogens from the flank, apical ectodermal ridge (AER) and zone of polarizing activity (ZPA) guide the pattern formation and establish the identity of emerging skeletal elements.^25,100–103^ Since E11.5, the forelimb mesenchyme condensation starts. The cartilage primordium is initially formed where the mesenchymal cells differentiate into chondrocytes at the core of condensation and into perichondrial cells immediately outside the condensation under the guidance of the master transcriptional factor SOX9.^104^ The round immature chondrocytes at the central portion of the cartilage primordia then stop proliferating and undergo morphological change into hypertrophic chondrocytes, whose volume increases around 20-fold.^105,106^ The distally located round chondrocytes however continuously proliferate and give rise to flattened stacked chondrocytes, which together with hypertrophic chondrocytes form the fetal growth plate.

*Prx1-Cre* marked all the mesenchymal cells in the developing limb bud, which gave rise to chondrocytes, perichondrial cells, periosteal cells, osteoblasts, and stromal cells, but not muscle cells.^107^ Similarly, *Sox9-Cre* labeled a more restricted population of the limb mesenchyme from E10.0, and Sox9^+^ progenitors gave rise to chondrocytes and osteoblasts.^108^ However, it is unclear whether a particular group of limb mesenchymal progenitors generate Sox9^+^ osteochondral progenitors.

### SSCs in endochondral bone formation

The hypertrophic chondrocytes of the fetal growth plate secrete Indian hedgehog (Ihh), which induces the expression of parathyroid hormone-related peptide (PTHrP) in the periarticular perichondrium that signals to PTHrP receptor in the pre-hypertrophic chondrocytes and delays Ihh production, and therefore a PTHrP-Ihh feedback is formed to maintain the growth plate structure.^81,83^ Besides this, Ihh directly targets to the perichondrial progenitors and commits their fate into Osx^+^ osteoblast precursors.^68,109^ Hypertrophic chondrocytes also secrete vascular endothelial growth factor (VEGF) and attract vascular invasion to form the primary ossification center.^110^

*Col2-Cre* marked perichondrial cells from E12.5, and Col2^+^ perichondrial cells gave rise to all the *Col1a1*-GFP osteoblasts at E14.5 and occupied the primary ossification center at E15.5.^67,68^ Absence of Runx2 is dispensable for the appearance of Col2^+^ perichondrial cells.^67^ *Osx-cre* marked perichondrial cells in a more restricted domain than *Col2-Cre* at E12.5. At E14.5, Osx^+^ cells resided in the inner perichondrium broader than Col1a1^+^ cells.^67^ Importantly, the perichondrial Osx^+^ pre-osteoblasts but not the Col1a1^+^ mature osteoblasts, translocated into the bone marrow along with the blood vessels in a pericyte-like fashion during development.^111^ These Osx^+^ progenitors transiently gave rise to trabecular osteoblasts, osteocytes and stromal cells, and disappeared along with longitudinal growth.^69^

A group of *Nes*-GFP^+^ cells were found in the perichondrium at E12.5, but these cells were endothelial. From E13.5, a group of nonendothelial CD31^−^*Nes*-GFP^+^ cells appeared in the innermost of the perichondrium and increased with endochondral bone formation. The emergence of CD31^−^*Nes*-GFP^+^ progenitors required Ihh and Runx2, and a portion of CD31^−^*Nes*-GFP ^+^ cells expressed *Osx.*^68^ Similar to Osx^+^ pre-osteoblasts, CD31^−^*Nes*-GFP^+^ cells invaded into the primary ossification center along the blood vessels. However, as *Nes*-GFP labeled both the endothelial and nonendothelial cells, *Nes-CreER* preferentially labeled the endothelial CD31^+^*Nes*-GFP^+^ cells.^68^ Thus, this marker seems not suitable for the tracing of osteoblast lineages. Embryonic Gli1^+^ progenitors also resided at perichondrium, articular cartilage surface, and Ranvier’s groove at E13.5, and contributed to primary ossification center at E18.5.^98^ To conclude, perichondrial cells participate in the formation of primary ossification center. It is likely that Col2^+^ cells give rise to CD31^−^*Nes*-GFP^+^ and Osx^+^ cells, which partially overlap.

Besides the contribution of perichondrial cells to the nascent bone marrow, growth plate hypertrophic chondrocytes transdifferentiate into osteoblast lineages and stromal cells.^112–114^ *Col2-Cre* not only labeled perichondrial cells but also growth plate chondrocytes since E12.5.^67^ A single tamoxifen injection at E13.5 to pulse chase the fate of *Col2-CreER* labeled cells further proved that Col2^+^ cells contributed to primary ossification center formation at E16.5 and postnatal day 0 (P0). Col2^+^ cells and descendants occupied both the primary ossification center and secondary ossification center (SOC) at P21.^67^ In fact, the rapid longitudinal bone growth during the fetal-neonatal period is supported by constant consumption of Col2^+^ chondroprogenitors, which generated short and multiclonal columns. The chondroprogenitors obtained the ability of self-renewal and formed large monoclonal columns only after the formation of SOC, along with the rise of a stem cell niche at the resting zone of the epiphyseal cartilage.^42^

As bone grows and vascular invasion progresses, it is possible that SSCs are established in the marrow cavity. Studies have uncovered different groups of SSCs located perivascular and support hematopoiesis postnatally.^39,60,78,94,115,116^ Though HSCs home to bone marrow around birth, whether the hematopoiesis-supporting cells are pre-established during fetal bone development is unclear.^117^ One interesting finding is the C-KIT^+^ mesenchymal cells.^118^ C-KIT ligand/C-KIT signaling is known to maintain HSCs in bone marrow. Postnatal C-KIT^+^ cells did not mark osteoblasts or adipocytes, but fetal C-KIT^+^ progenitors gave rise to bone and bone marrow stroma. The chondrocytes, pre-osteoblasts and bone marrow stromal cells at E16.5 were labeled when *Kit*^*MerCreMer*^*;R26R-*tdTomato mice received tamoxifen at E12.5 and E14.5.^118^ However, it is unclear whether the bone marrow C-KIT^+^ cells are derived from perichondrial or growth plate chondrocytes, or re-established in bone marrow.

### SSCs in postnatal long bone growth

From neonatal to early adolescence, the growth plate chondroprogenitors are the major source of osteoblasts and stromal cells. Tracing the fate of early postnatal Col2-expressing cells by a single tamoxifen injection to *Col2-Cre*^*ER*^*;R26R-*tdTomato mice at P3 showed that Col2^+^ cells gave rise to osteoblasts and stromal cells in the metaphysis and epiphysis, but not diaphysis at 1 month of age. These Col2^CreER^-P3 cells continued to become osteoblasts, stromal cells and adipocytes for 1 year.^67^ Besides *Col2*, genes involved in cartilage development including *Sox9* and *Acan*, are able to mark growth plate chondroprogenitors. Both *Sox9-Cre*^*ER*^ and *Acan-Cre*^*ER*^ marked comparable populations of osteoblasts and stromal cells at least up to 6 months when receiving a single dose of tamoxifen at P3.^67^

More solid evidence that the growth plate harbors SSCs comes from the study of the PTHrP^+^ chondrocytes.^41^ A group of PTHrP-mCherry^+^ chondrocytes that were less proliferative appeared at the resting zone of growth plate at P3 and rapidly increased in number between P6 and P9. Lineage tracing using *Pthrp-Cre*^*ER*^*;R26R-*tdTomato mice and *Pthrp-Cre*^*ER*^*;R26R-*Confetti mice proved that a single PTHrP^+^ chondrocyte gave rise a longitudinal column. Long-term tracing showed that a fraction of PTHrP^+^ chondrocytes differentiated into osteoblasts and stromal cells, but not adipocytes in bone marrow. Besides, flow cytometry analysis showed PTHrP-mCherry^+^ resting chondrocytes exhibited immunophenotype that partially overlapped with the CD51^+^CD90^−^CD105^−^CD200^+^ mSSCs. The appearance of SOC was crucial for the self-renewal and clonality of PTHrP^+^ chondrocytes, as P12 PTHrP^+^ cells, but not P9 PTHrP^+^ chondrocytes, survived rounds of passage in vitro.^41^ This is in accordance with the finding that growth plate SSC niches emerged with the formation of SOC.^42^ Recently, a group of FoxA2^+^ SSCs residing at the top compartment of the resting zone and immediately adjacent to SOC were identified.^119^ FoxA2^+^ SSCs were distinct population from PTHrP^+^ cells and possessed higher clonogenicity and longevity. At early postnatal stage, FoxA2^+^ cells generated SOC bone cells and growth plate chondrocytes. After P28, FoxA2^+^ cells only contributed to chondrogenic lineages. The major function of FoxA2^+^ SSCs was to maintain growth plate turnover and regeneration after injury.

Besides the growth plate chondroprogenitors that generate bone components, several types of SSCs or lineage-restricted progenitors emerge around the highly vascularized marrow space just beneath the growth plate and function as an important source for osteoblasts and marrow stromal cells.^68,69,97,98^ Neonatal Osx^+^ cells were located at primary spongiosa and around the cortical bone. A single tamoxifen injection to *Osx-Cre*^*ERT2*^*;R26R-*tdTomato mice at P5 labeled Tomato^+^ cells that gave rise to osteoblasts and osteocytes after 3 weeks. Surprisingly, Tomato^+^ cells continuously gave rise to long-lived marrow stromal cells at least for 32 weeks.^69^

Postnatal Gli1^+^ cells were observed at the chondro-osseous junction beneath the growth plate, which gave rise to *Col1a1*-GFP osteoblasts.^98^ Gli1^+^ cells highly expressed Pdgfra and Runx2, but not Lepr and Osx at 1 month of age. At 6 months, half amount of the Gli1^+^ cells were Lepr^+^, indicating postnatal Gli1^+^ cells gave rise to Lepr^+^ cells with age. Both hedgehog and Wnt signaling were required for the osteoblastic fate commitment of Gli1^+^ cells. However, Gli1^+^ were mainly enriched in young postnatal mice and dramatically diminished with age.^98^

Gremlin1 also labeled a group of postnatal non-perivascular osteochondroreticular (OCR) stem cells that located at the primitive mesenchyme and primary spongiosa at P1.^97^ Grem1^+^ cells gave rise to chondrocytes, osteoblasts, marrow stromal cells but not adipocytes. Four weeks after a single injection of tamoxifen to *Grem1-Cre*^*ERT*^*;R26-*tdTomato*;col2.3-GFP mice*, red fluorescence was observed in a majority of bone and chondrocytes within the metaphysis and epiphysis, but not in diaphysis. DTA-driven Grem1^+^ cells ablation significantly diminished postnatal bone formation in the femoral epiphysis. Compared with *Nes*-GFP^+^ cells, Grem1^+^ cells exhibited a higher CFU capacity. Transcriptional profiling of Grem1^+^ cells indicated higher osteochondrogenic potential and inhibition of adipogenesis.^97^

Aforementioned SSCs and progenitors are marked by promoters of the transgenes that are actively involved in skeletal development. This unavoidably poses a question that whether a panel of transgenes can mark SSCs once the genes play an essential role in bone development. One intriguing example is the Hox11 paralogous genes that specifically participate in zeugopod pattern formation.^120,121^ By lineage fate-mapping of *Hoxa11*-EGFP and *Hoxa11-Cre*^*ERT2*^-expressing cells, members in Wellik Laboratory demonstrated that Hox11 lineage-positive cells were sufficient to give rise to all the skeletal components regionally restricted to zeugopod throughout life, highlighting SSCs arise from the earliest stages of bone development and can be labeled by a region-specific marker.^120,121^ However, *Dlx5-Cre*^*ER*^ only transiently labeled a group of cells and made little contribution to bone components, despite an essential role of Dlx5 in development.^41^ Therefore, it remains to be answered why only some promoters label the authentic SSC populations.

### Skeletal stem cells in long bone maintenance

#### The fate transition of short-term progenitors to long-lived SSCs

The contribution of chondrocytes to bone marrow osteoblasts gradually decreases with the completion of long bone growth. Adult bone marrow SSCs become the major source of osteoblasts instead. Aforementioned studies have shown that the short-term perinatal Col2^+^ chondrocytes and Osx^+^ cells gave rise to long-lived bone marrow SSCs, indicating a reestablishment of cellular identity from osteochondral progenitors.

Using dual-recombinase fate-mapping system, Shu et al. captured the transition of chondrocytes to adolescent bone marrow SSCs.^77^ They first constructed *Col2-Cre;Lepr*^*dreER*^*;R26*^*LSL-*ZsGreen^*;R26*^*RSR-*tdTomato^ mice, in which the *Col2-Cre*-derived cells expressed ZsGreen and *Lepr-Dre*-derived cells expressed Tomato. After tamoxifen induction, a great portion of ZsGreen^+^ cells and Tomato^+^ cells overlapped, indicating the genetic relation of Col2^+^ cells and Lepr^+^ cells. A mutually exclusive tracing system (*Col2*^*dre*^*;Lepr*^*cre*^*;IR* mice) was then developed, in which the first recombination prevented the other. The frequency of *Lepr-Cre*-marked ZsGreen^+^ cells was greatly reduced, accounting for only 11% in single-recombinase *Lepr*^*cre*^*;IR* mice, rigorously demonstrating Col2 lineage sat upstream of Lepr lineage. Interestingly, the osteoblasts formed by Col2^+^ cells and Lepr^+^ cells exhibited a spatially separate distribution, where the Lepr-derived osteoblasts first emerged in diaphysis and progressively spread to metaphysis. The molecular mechanism regulating this transition remains to be answered. Nonetheless, growth plate Col2^+^Col10a1^+^ chondrocytes only gave rise to ~58% of Lepr^+^ cells. About 12% of the Lepr^+^ cells were derived from Col2^+^Col10a1^−^ periosteal cells and the origin of the remaining 30% is unknown.^77^ One study compared the functional difference of growth plate (Col10a1^+^) and non-growth plate (Col10a1^−^) derived Lepr^+^ CFU-Fs. They exhibited similar osteogenic and adipogenic capacities in vitro. Surprisingly, when transplanted underneath renal capsule, the Col10a1^+^ CFU-Fs formed a complete ossicle with bone marrow whereas Col10a1^−^ CFU-Fs only formed bone.^122^

Bone marrow LepR^+^ cells only comprised 0.3% of bone marrow cells, but accounted for 94% of CFU-Fs.^64^ LepR^+^ cells arose postnatally, residing around sinusoids and arterioles. LepR^+^ cells expressed MSC markers Prx1, PDGFRα, CD51 and *Nes*-GFP^low^, and were highly enriched for hematopoiesis-supporting factor *Scf*-GFP and *Cxcl12*-DsRed. Lineage tracing showed LepR^+^ cells gave rise to a majority of osteoblasts and adipocytes in the bone marrow from 8 weeks of age. LepR^+^ cells remained quiescent physiologically, but were rapidly activated in response to irradiation and bone fracture. Transplantation of LepR^+^ cells into the bone marrow of sub-lethally irradiated mice supported bone, cartilage and adipocytes formation.^64^

A major difference between Col2^+^/Acan^+^ osteoprogenitors and Lepr^+^ SSCs lies in their function in supporting bone longitudinal growth and appositional remodeling, respectively.^77^ Loss of Runx2 in perinatal Acan^+^ cells retarded bone growth while in LepR^+^ cells reduced cortical bone thickness. Mechanical stimulus such as running only improved the osteoblastic differentiation of perinatal Acan^+^ cells but not adult LepR^+^ cells. Importantly, tamoxifen-induced expression of *LepR-Cre*^*ER*^ cells at P1-P3 only gave rise to ~28% of all bone marrow LepR^+^ cells after 2 months, and these perinatal LepR^+^ cells made little contribution to bone formation, which makes *LepR-Cre/LepR-Cre*^*ER*^ an ideal system to study postnatal adult bone marrow skeletal stem cells.^77^

*Cxcl12-Cre*^*ER*^ labeled a dormant subset of *Cxcl12*-GFP^high^ perisinusoidal stromal cells that resided in the central marrow and ubiquitously expressed Lepr. In response to cortical bone injury or marrow ablation, *Cxcl12-Cre*^*ER*^-expressing cells transformed into skeletal stem-like cells via Wnt signaling, and contributed to bone regeneration. This transition indicates the cellular plasticity of *Cxcl12-Cre*^*ER*^-expressing cells.^123^

#### Bone marrow SSCs and niches

Apart from the contribution to osteo-lineages, one major function of adult bone marrow SSCs is to support hematopoiesis.^60,94,115,124,125^ Perivascular stromal cells, including endothelial cells, LepR^+^ cells, CXCL12-abundant reticular (CAR) cells and *Nes*-GFP^+^ cells, are implicated in hematopoietic stem and progenitor cell maintenance by secreting CXCL12 or stem cell factor (SCF).^78,94,126^

Nestin was first identified to label bone marrow SSCs, but different *Nestin* transgenes, including *Nes*-GFP, *Nes*-Cherry, *Nes-Cre,* and *Nes-Cre*^*ER*^, seemed to label different groups of perivascular stromal and endothelial cells.^60,78^ It is now confirmed that *Nes*-GFP^+^ cells labeled perivascular SSCs and constituted HSC niche component. HSCs transplanted into lethally irradiated mice homed to *Nes*-GFP^+^ cells and ablation of Nes^+^ cells impaired HSC content.^78^ However, other studies reported that SCF or CXCL12 deletion in *Nes-Cre/Nes-Cre*^*ER*^-expressing cells hardly affect HSCs frequency and function.^60^

LepR^+^ cells largely overlapped with CAR cells and constituted the HSCs niche. Knockout of *Scf* from LepR^+^ cells but not *Col2.3-Cre*-expressing osteoblasts depleted quiescent HSCs from bone marrow.^60^ *Ebf3-Cre* also labeled CAR/LepR^+^ cells and Ebf3^+^ cells were self-renewable. Interestingly, *Ebf3* deletion in *LepR-Cre*-expressing or *Prx1-Cre*-expressing cells led to osteosclerosis and HSCs depletion, indicating the important role of Ebf3 in maintaining stemness and HSCs niches.^93^

Endosteal niches harbored osteoprogenitors instead. It is noteworthy to mention that different from fetal bone where the perichondrial Osx^+^ osteoblast precursors gave rise to mature osteoblasts and osteocytes, adult osteocalcin^+^ osteoblasts and Osx^+^ pre-osteoblasts turned over rapidly, and were continuously replenished by SSCs or lineage-committed progenitors.^127^ The *myxovirus resistance-1 (Mx1)-Cre* labeled osteo-lineage-committed progenitors at endosteal bone that efficiently gave rise to osteoblasts but not adipocytes or chondrocytes in vivo, though labeled cells are capable of multi-lineage differentiation in vitro.^127^ Interestingly, by conditional deleting CXCL12 in different niches, two companion work demonstrated that osteo-lineage-committed cells supported lymphoid progenitors.^94,115^ In support of these findings, a recent study identified a subpopulation of periarteriolar LepR^+^osteolectin^+^ osteogenic progenitors. These cells were fast-dividing and short-lived, which was different from the quiescent nature of self-renewable LepR^+^ cells. LepR^+^osteolectin^+^ cells were responsive to mechanical stimulus and supported lymphopoiesis.^128^ Moreover, this study indicated that not all LepR^+^ perivascular cells are long-lived SSCs. Some subgroups of LepR^+^ population are indeed committed progenitors.

#### Bone marrow adipocytes precursors

Bone marrow adipocytes share a mesenchymal origin with osteoblasts.^129,130^ The fate decision of SSCs is tilted toward adipocytes during ageing or under pathological conditions such as myeloablation, high-fat diet and caloric restriction.^22,86,129,131^ As discussed above, many transgenes (e.g. *Prx1-Cre*, *Osx-Cre*, *LepR-Cre*) are able to mark marrow adipocytes.^64,69,132^ Importantly, *Adiponectin-Cre*^*ER*^ *(Adipoq-Cre*^*ER*^*)* was reported to mark a subpopulation (~5%) of *LepR-Cre*-expressing cells that preferentially differentiated into adipocytes.^133^

Using flow cytometry and transplantation assays, Ambrosi et al. plotted the marrow adipocytic lineage differentiation trajectory.^72^ A population of multipotent perivascular CD45^−^CD31^−^Sca1^+^CD24^+^ progenitors gave rise to lineage-committed CD45^−^CD31^−^Sca1^+^CD24^−^ adipogenic progenitor cells (APCs), which generated a more mature CD45^−^CD31^−^Sca1^−^Zfp423^+^ pre-adipocytes (preAd). Meanwhile, a group of CD45^−^CD31^−^Sca1^−^PDGFRα^+^ osteochondrogenic progenitors could be differentiated from CD45^−^CD31^−^Sca1^+^CD24^+^ progenitors in parallel.^72^

Likewise, a population of bone marrow adipogenic lineage precursors (MALPs) that situated downstream of SSCs and upstream of lipid-laden adipocytes were identified by Qin Lab.^134,135^ They profiled the *Col2-Cre;R26R-*tdTomato-expressing cells using scRNA-seq, and identified a cluster of MALPs that were efficiently labeled by *Adipoq-Cre/Adipoq-Cre*^*ER*^ as stromal cells and pericytes since newborn. These cells were non-proliferative, non-lipid-laden (Perilipin^−^) and protruded to form a three-dimension network. MALPs could differentiate into lipid-laden Adipoq^+^Perilipin^+^ mature adipocytes. Ablation of MALPs with diphtheria toxin impaired blood vessels but increased bone formation in *Adipoq-Cre;DTR* mice, without significant influence on hematopoiesis.^134^ Long et al. reported the *Col10a1-Cre*-derived cells in the bone marrow were not pericytes, though they were associated with blood vessels.^122^ As growth plate Col10a1^+^ cells are immediately downstream of Col2a1^+^ cells, why do *Adipoq-Cre*-marked populations function as pericytes needs further clarification. More importantly, a recent study constructed the *Adipoq-Cre;mTmG* mice and traced the fate of Adipoq^+^ cells from 1 month to 9 months. They surprisingly identified that Adipoq^+^ cells contributed to bone formation with age. In 1-month-old mice, GFP-expressing cells were restricted to marrow stroma. In 9-month-old mice, the ratio of GFP-expressing osteocytes to all osteocytes almost reached 40%. This study indicated that at least a subset of *Adipoq-Cre*-expressing cells were bipotent.^136^

## Skeletal stem cells in craniofacial bones

### Origin of craniofacial SSCs

The craniofacial bones originate from the paraxial head mesoderm and the cranial neural crest cells (CNCCs).^138,139^ The anterior skull bones are derived from the CNCCs, whereas the posterior part originates from the paraxial head mesoderm.^138^ CNCCs are highly plastic compared to the trunk neural crest cells.^140^ CNCCs give rise to a majority of mesenchymal progenitors of facial cartilage and bone elements. *Twist1* gene is activated upon delamination in the cranial compartment and directs CNCCs toward a mesenchymal fate. Intriguingly, sustained overexpression of *Twist1* was sufficient to reverse the developmental program of the trunk crest to a mesenchymal route.^140^ Schwann cell precursors (SCPs) are direct progeny of neural crest and generate osteochondral progenitors in facial region.^141^ The contribution of individual CNCC to facial components and the spatial organization of the ectomesenchyme has been revealed.^142^ During early outgrowth and shaping of the facial regions, organization is mainly driven by oriented cells division, allocation and relocation but minimal individual cell migration, which indicates a conserved program of facial outgrowth.^142^

### SSCs in alveolar bone

Distinctive from long bone, our understanding of niches and stem cells in alveolar bone is lacking. We have plotted a single-cell atlas of mouse mandibular alveolar bone and identified a microenvironment with a higher portion of mature immune cells compared with long bone marrow. A high level of Oncostatin M (Osm) was found in alveolar bone marrow monocytes/macrophages, which promoted osteogenic differentiation and inhibited adipogenic differentiation of alveolar MSCs.^143^ Further, we have identified a tissue-resident LepR^+^ subpopulation in alveolar bone of the adult mice, which contributed to socket healing of tooth extraction. The biological behavior of LepR^+^ cells were regulated by PTH/PTH1R signaling.^116^

SM22α-lineage niche cells have been recently found to regulate regeneration of alveolar bone.^144^ They were quiescent physiologically and expanded following bone injury. Moreover, SM22α-lineage niche cells did not act as stem cells and instead regulated alveolar bone regeneration via PDGFRβ-triggered hydrogen sulfide (H_2_S) production.^144^

### Cranial suture stem cells

Adult Gli1^+^ suture stem cells (SuSCs) were responsible for maintenance and injury repair of cranial bones.^145^ Ablation of Gli1^+^ SuSCs caused craniosynostosis and growth retardation of mouse skull.^145^ The bone regeneration was faster when injury site was located closer to the suture, supporting that Gli1^+^ SuSCs are the source for the cranial bone regeneration.^146^ Craniosynostosis of *Twist1*^*+/*−^ mice model showed reduced number of Gli1^+^ SuSCs, suggesting that diminished SuSCs may account for suture closure.^145^ A translational research showed Gli1^+^ SuSCs-based functional cranial suture regeneration corrected skull deformity along with cognitive defect of *Twist1*^*+/*−^ mice model. The regenerated suture created a niche for endogenous SuSCs migration to support cranial bone homeostasis and injury repair.^147^

Distraction osteogenesis is adopted to correct underdevelopment of the skull and maxilla. In mouse suture expansion model, Gli1^+^ SuSCs contributed to bone remodeling upon tensile force loading. Reduction of Wnt signaling decreased such contribution.^148^

Axin2-expressing population within cranial suture were long-term self-renewal and clonogenic. Axin2^+^ SuSCs contributed to injury repair of cranial bones in a cell autonomous manner.^149^ CD51^+^CD200^+^ cells were confirmed as resident SSCs in cranial sutures.^150^ Reduced CD51^+^CD200^+^ population was found in posterior frontal suture closure and craniosynostosis. Wnt activation increased CD51^+^CD200^+^ cells and rescued craniosynostosis by preventing suture closure.^150^

Prx1-expressing cells were identified in the cranial suture mesenchyme at P12 of *Prx1-Cre*^*ER*^*;EGFP* mouse model.^151^ Prx1^+^ cells were identified in the posterior frontal sutures, coronal sutures, sagittal sutures, and lambdoid sutures by intravital microscopy.^152^ Prx1^+^ cells progressively reduced from 8-week-old mice to 32-week-old mice. Ablation of postnatal Prx1^+^ lineage using DTA showed that they were required for cranial bone defect regeneration, but not for calvarial growth.^152^

### Stem cells in periodontal ligament

Postnatal MSCs of human periodontal ligament was firstly isolated in 2004.^153^ CD45^−^Ter119^−^Tie2^−^CD51^+^Thy^−^6C3^−^CD105^−^CD200^+^ mSSCs were recently sorted out from mouse periodontal ligament (mPDL) tissue.^154^ mPDL-derived SSCs exhibited clonogenic, cementogenic and odontogenic capacity.^154^

Gli1^+^ cells were multipotent stem cells within mouse molar PDL and contributed to turnover of periodontal supporting tissue.^155^ PDL Gli1^+^ cells resided around the neurovascular bundle especially in apical region of the mouse molar. PDL Gli1^+^ cells were responsive to Wnt signaling and removal of occlusal force inhibited Gli1^+^ cells activation by upregulating sclerostin.^155^ Mechanoresponsive property of Gli1^+^ cells was supported by Yap, a classical mechano-transduction factor.^156^ Using *Gli1*^*LacZ/+*^ and *Gli1-Cre*^*ERT2*^*;R26R*^tdTomato^ mouse models, study showed cellular cementoblasts did not express Gli1 (β-galactosidase^-^), and Gli1^+^ cells contributed to cementum formation (Tomato^+^). Ablation of Gli1^+^ cells using DTA reduced cementogenesis. Reduction of Wnt/β-catenin signaling in Gli1^+^ cells decreased cementum formation postnatally.^157^ PDL is responsive to Wnt signaling.^158^ Axin2^+^ cells were identified as PDL stem cells supporting the homeostasis of the periodontium. Orthodontic tension force increased the number of PDL Axin2^+^ cells.^159^ In response to tooth extraction, Axin2^+^ cells contributed to bone healing, and healing accelerated when WNT3A protein was applied.^160^ Axin2^+^ lineage also contributed to cementum formation at postnatal rapid growth period.^161^ Comparatively, K14^+^ epithelial cells were initially active at early development and reduced in number at postnatal period from P28 to P56. Ablation of Axin2^+^ lineage caused cementum hypoplasia. Activation of Wnt/β-catenin signaling in Axin2^+^ lineage promoted cellular cementogenesis.^161^ CD90^+^ cells also gave rise to cementoblasts during postnatal development. During adult homeostasis, however, only Axin2^+^ contributed to cementogenesis, whereas CD90^+^ cells were reactivated in mice with periodontitis.^162^

Postnatal Prx1^+^ cells were found within the PDL of mouse incisor and ablation of Prx1^+^ lineage at P3 led to PDL enlargement at P21.^163^ Prx1^+^ cells contributed to regeneration of non-critical size periodontal defect. Prx1^+^ cells were also identified within human PDL.^163^ Deletion of *Pth1r* using *Prx1-Cre* arrested the eruption of the mandibular incisor.^164^ Lineage tracing of postnatally *Prx1-Cre;R26R-*tdTomato mouse showed Prx1^+^ cells were mainly restricted to the first molars, in which PDL Prx1^+^ cells contributed to almost all types of mesenchymal cells. PDL reconstruction of the transplanted molar required Prx1^+^ cells from the alveolar bone of the recipient mice.^165^

We recently constructed a *LepR-Cre*^*ER*^;tdTomato mouse model and identified a subset of LepR^+^ cells within the PDL region. Such LepR^+^ subsets were specifically located adjacent to blood vessels of the PDL and contributed to root furcation bone healing when PDL injury was induced (unpublished data). We also found a Ctsk^+^ subpopulation within the PDL using *Ctsk-Cre;*tdTomato mice. Ctsk^+^ cells were present throughout the entire PDL including furcation area and apical area. Ctsk^+^ cells also contributed to cementum and alveolar bone development, which share a developmental origin with PDL (unpublished data).

### Stem cells in Schneiderian membrane

The contribution of the sinus membrane to bone regeneration is widely concerned. Postoperative new bone formation was reported after maxillary sinus augmentation, suggesting pluripotent stem cells exist in the sinus membrane.^166^ In an experiment by Helms Lab, the posterior paranasal sinus in a preclinical mouse model served as a recipient site for autografts or bone graft substitutes, and autograft indicated faster new bone formation rate. Internal periosteum-derived Wnt-responsive progenitors of the maxillary bone was found to contribute to new bone formation.^167^

**Fig. 2:**
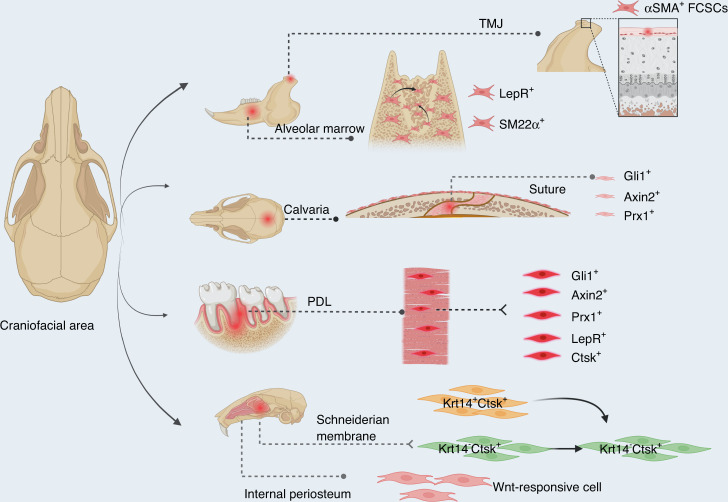
Schematics of craniofacial progenitors for bone formation. Craniofacial region harbors various cell sources. From the latest literatures and our research, the indicated cell sources play crucial roles in craniofacial bone formation and healing

A subset of Krt14^+^Ctsk^+^ cells have been identified as osteoprogenitors, which contribute to homeostasis and injury-induced osteogenesis of maxillary sinus floor. Such subset exhibited both epithelial and mesenchymal properties, and specifically played a role in bone regeneration after maxillary sinus floor lifting. Lineage tracing with dual recombinases showed that descendants of Krt14^+^Ctsk^+^ progenitors, which are Krt14^−^Ctsk^+^, underwent robust osteogenesis. Similarly, a Krt14^+^Ctsk^+^ subset was found in human Schneiderian membrane as well (Fig. 2).^168^

### Stem cells in other craniofacial tissues

A stem cell niche inhabits in tooth.^169^ Stem cells from human exfoliated deciduous teeth were identified.^170^ Stem cells in dental pulp and from oral mucosa were characterized and reported.^171,172^ Pericyte-derived and nonpericyte-derived MSCs altogether contributed to tooth growth and repair.^173^ A small population of Gli1^+^ slow-cycling MSCs supported by neurovascular bundle was identified to contribute to mouse incisor continuous growth.^174^ Proteolipid protein (PLP) was also reported to label slow-cycling cells in mouse incisor and contributed to a proportion of odontoblasts, suggesting the glial origin of dental pulp of mouse incisor.^175^

Stem cell-based temporomandibular joint regeneration is extensively studied.^176–178^ Potential of various human stem cells to regenerate TMJ has been explored and in vivo studies are expected.^176^ Notably, fibrocartilage stem cells (FCSCs) at the superficial zone of the condylar cartilage were identified with *αSMA-Cre*^*ERT2*^ and they generated and maintained the cartilage structure. A single FCSC was sufficient to form cartilage anlage, and recapitulated endochondral ossification to form bone and support hematopoiesis in vivo.^179^ Moreover, FCSCs could be isolated from human TMJ cartilage, suggesting the translational potential for condylar cartilage treatment of TMJ disorders.^180,181^

## Skeletal stem cells in periosteum

### Periosteal SSCs in long bone

**Fig. 3:**
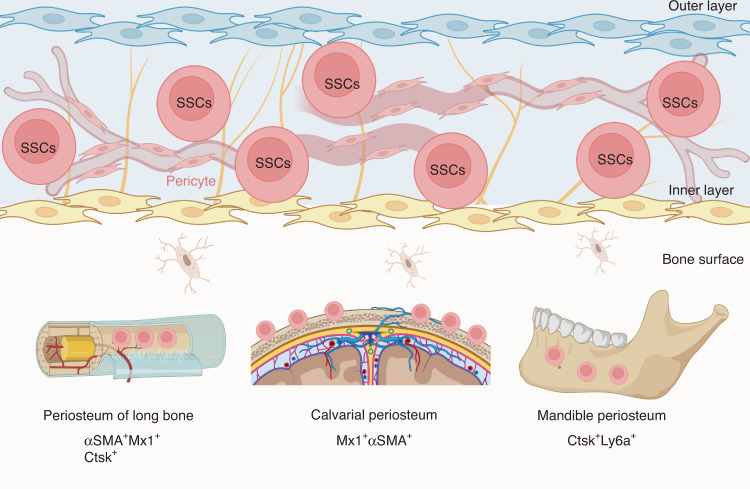
Skeletal stem cells in periosteum. Periosteum of long bones, calvaria, and mandible harbors SSCs that participate in bone development and injury repair

Periosteum is derived from outer layer of embryonic perichondrium, and harbors SSC niches that support cortical bone formation and architecture, and bone fracture repair postnatally (Fig. 3).^71,99,182–185^ Periosteal cells displayed an embryonic *Prx1-Cre*-expressing mesenchymal origin and were not derived from vascular invasion.^186^ *Ctsk-Cre* marked periosteal heterogenous mesenchymal cells despites its ability to label endosteal osteoclasts.^71,187^ FACS analysis divided CTSK-mGFP cells into three groups, the CD200^+^CD105^−^ periosteal stem cells (PSCs), CD200^−^CD105^−^ periosteal progenitor 1 (PP1) cells, and CD105^+^CD200^variable^ periosteal progenitor 2 (PP2) cells. PSCs first appeared at perichondrium of E14.5 femur and displayed self-renewal ability and clonal multipotency. PSCs generated the whole spectrum of CTSK-mGFP cells after serial transplantation assays in mammary fat pad. Therefore, PSCs are bona fide SSCs. Interestingly, PSCs did not support hematopoiesis and directly differentiated into osteoblasts via intramembranous ossification at physiological condition or when transplanted under the kidney capsule. However, PSCs remained plastic and contributed to fracture callus formation via both intramembranous and endochondral bone formation. Blocking the osteogenic ability of *Ctsk-Cre*-expressing cells via knockout of *Osx* dramatically impaired the cortical bone formation and fracture repair.^71^

*Ctsk-Cre*-mediated deletion of *liver kinase b1* (*Lkb1*) led to osteosarcoma via activation of mammalian target of rapamycin complex 1 (mTORC1).^188^ Besides, *Ctsk-Cre* also labeled progenitors at the perichondrial groove of Ranvier, a recognized niche for stem cells.^189,190^ Knockout of *Ptpn11*, genes encoding tyrosine phosphatase SHP2, in *Ctsk-Cre*-expressing cells caused metachondromatosis due to overactivation of hedgehog signaling at the perichondrial groove of Ranvier.^189^ Thus, these studies indicate that postnatal periosteal/perichondrial *Ctsk-Cre*-expressing cells are an origin of tumor when dysregulated. Moreover, *Ctsk-Cre* labeled PSCs played an indispensable role in maintaining postnatal growth plate structure and prolonged longitudinal bone growth by secreting IHH. Interestingly, IHH production from developing growth plate progressively reduced with age, whereas postnatal PSCs-derived IHH supported the proliferation of growth plate resting zone SSCs, indicating a crosstalk between periosteal and growth plate stem cells.^191^

αSMA was identified to mark periosteal progenitors that differentiated into osteoblasts and chondrocytes during fracture healing.^95^ Ortinau et al. further identified a group of Mx1^+^αSMA^+^ periosteal cells. Around 80% of Mx1^+^αSMA^+^ cells expressed SSC immunophenotypic markers (CD105^+^CD140a^+^) whereas only ~10% of Mx1^−^αSMA^+^ cells were CD105^+^CD140a^+^. Mx1^+^αSMA^+^ periosteal cells were long-lived population and continuously gave rise to osteoblasts. Moreover, Mx1^+^αSMA^+^ periosteal cells expressed the C–C Motif Chemokine Ligand 5 (CCL5) receptor CCR3 and CCR5, and migrated in a CCL5-dependent manner to bone injury site.^99^ Nevertheless, the relationship between CTSK-mGFP PSCs and Mx1^+^αSMA^+^ SSCs is currently unknown.

### Periosteal SSCs in calvarial bone

In a mouse model of calvarial injury, Mx1^+^αSMA^+^ cells migrated to and resided in the center of the injury site, which differentiated into Mx1^+^αSMA^−^ bone cells. Administration of CCL5 promoted migration of Mx1^+^αSMA^+^ periosteal cells to the injury site for calvaria repair (Fig. 3).^99^

### Periosteal SSCs in mandible

By mapping the transcriptional landscape of the human mandible periosteum, a Ctsk^+^ subset was identified. Further identification presented Ctsk^+^Ly6a^+^ subset as periosteal progenitors that were activated during injury repair (Fig. 3).^192^ The periosteum SSCs are regulated by schwann cells in a paracrine manner.^193^ Myelin PLP^+^ schwann cells played a role in tissue repair.^194–196^ By constructing an inferior alveolar nerve (IAN) denervation mouse model, study showed adult schwann cell paucity reduced contribution of SSCs to mandibular healing.^193^

Mandible periosteal SSCs are mechanoresponsive. Members in Longaker Lab analyzed the chromatin and transcriptional profile of SSCs during mandible distraction osteogenesis, and found an activation of CNCC-like program in SSCs in focal adhesion kinase (FAK)-dependent manner. This study indicated that during mouse mandible regeneration, SSCs could reverse to developmental stage and regained a CNCC-like identity.^197^

## Single-cell landscape of skeletal components

Single-cell transcriptomics have revealed a more complex landscape of the skeletal components.^198,199^ The differentiation trajectory of progenitors during bone development and homeostasis was plotted, unveiling the heterogeneity of SSCs.

### Long bone at single-cell resolution

Human embryonic SSCs ontogeny was dissected by scRNA-seq.^90^ Cells in limb buds at 5 weeks post conception (WPC) were divided into 10 subsets, of which 4 subsets were of mesenchymal origin. They were limb bud mesenchymal subsets 1–3 (LBM1–3) that were *PRRX1*^high^ and differentially expressed *PDGFRA*, and osteochondrogenic progenitors (OCPs) that were *PRRX1*^+^, *SOX9*^low^ and *PDGFRA*^high^. LBM1 and LBM2 were distally positioned, preferentially expressing 5′ *HOX* genes, while LBM3 and OCPs were at proximal end, preferentially expressing 3′ *HOX* genes. LBM2 was highly proliferative, enriched with metabolic process and closely associated with AER, probably annotating the progress zone. Nevertheless, a comparative analysis showed a much lower portion of AER cluster and lack of LBM2 in mouse hindlimb buds at E11.5, indicating an accelerated maturation of mouse limb bud. Embryonic long bone was already formed at 8 WPC, and the osteochondral lineage was divided into 7 subsets, including three OCP subsets. They were the limb bud-derived mesenchymal cells (LBDMCs), BMSCs and embryonic skeletal stem/progenitor cells (eSSPCs). LBDMCs highly expressed *TWIST2* and sat upstream of BMSCs (*CXCL12*^high^ and *PDGFRA*^high^) and eSSPCs, a novel perichondrial subset that was highly enriched with FOXP1/2/4 regulons. The PDGFRA^low/–^PDPN^+^CADM1^+^ cells could enrich the self-renewable eSSPCs and gave rise to osteochondral lineages in vivo, but did not reconstitute hematopoietic environment. Besides, the eSSPCs population could be identified in mouse E15.5 long bones.^90^

Kelly et al. plotted the single-cell landscape of mouse embryonic hindlimb development.^200^ Whole hindlimb tissue was dissociated for scRNA analysis at four time points (E11.5, E13.5, E15.5. E18.5). Seven clusters were identified across all time points, representing cartilage, bone/tendon, skin, muscle, blood, vasculature and cell cycle. An unsupervised algorithm predicting the developmental trajectories showed a branch of musculoskeletal precursors arose at E11.5 and the vasculature branch arose at last.^200^

Single-cell transcriptomics helped to prove the hypothesis that non-mitotic hypertrophic chondrocytes dedifferentiate into skeletal stem and progenitor cells, which then generate osteoblasts and adipocytes in the bone marrow.^122^ *Col10a1-Cre;R26R-*tdTomato-marked hypertrophic chondrocytes and progenies at E16.5 and 2 months of age were sorted for scRNA-seq and in vivo validation. At E16.5 when primary ossification center starts to form, scRNA-seq identified an intermediate cluster that linked hypertrophic chondrocytes to osteoblasts, and exhibited SSCs-like properties. Similarly, 3 clusters of marrow-associated cells derived from hypertrophic chondrocytes at 2 months of age exhibited a more primitive state compared with osteoblasts, and highly expressed SSC markers *Lepr*, *Gremlin1*, *Pdfgra*, *Cxcl12* and *Pdgfrb*. Nearly 25%–45% of marrow SSCs were derived from hypertrophic chondrocytes, and perichondrium/periosteum might constitute the other portion of SSCs in bone marrow.^122^

In align with this endochondral ossification process, a group of mesenchyme-derived fatty acid binding protein 5 (FABP5)-expressing septoclasts that aided in matrix degradation and chondrocyte phagocytosis were reported by Adams Laboratory.^201^ FABP5^+^ cells were located at the chondro-osseous border and expressed PDGFRα and PDGFRβ. Single-cell analysis of the *PDGFRα*-GFP cells identified a small portion of cells that were FABP5^+^ and Mmp9^+^. This FABP5^+^ cluster emerged from and was closely associated with the PDGFRβ^+^ metaphyseal MSCs populations.^201^

Postnatal bone marrow consists of highly complicated environment with mesenchymal and hematopoietic cells forming different niches that guarantee the physiological function of hematopoiesis and bone remodeling. Wolock et al. isolated bone marrow nonhematopoietic and non-endosteal stromal cells from adult mice for scRNA-seq, and confirmed that MSCs committed fate into either adipocytes or osteoblasts/chondrocytes. Interestingly, cultured stromal cells exhibited a different landscape with freshly isolated stromal cells, with a larger proportion of cultured cells resembling osteoblast progenitors.^202^

A more detailed clustering of BMSCs from P21 bone marrow was exploited by Sivaraj et al.^137^ Seven subclusters were identified, including diaphyseal MSC 1 and 2 (dpMSC1, dpMSC2), metaphyseal MSC (mpMSC), proliferating BMSC (P-BMSCs), osteoprogenitor cells 1 and 2 (OPC1, OPC2), and osteoblasts (OBs). Trajectory analysis indicated mpMSCs were at the center and gave rise to other cell types. However, *Lepr* expression was detected only in cluster dpMSC1 and dpMSC2, but not in mpMSC. Diaphyseal *LepR*^*+*^ cells were located around sinusoids and highly express *Kitl* and *Cxcl12*. A lineage tracing using *LepR-Cre;R26R-*tdTomato mice showed that metaphyseal tdTomato^+^ cells did not express dpMSC immunophenotypic markers ESM1 and PDGFRβ, indicating the heterogeneity of LepR^+^ populations.^137^ In an integrated analysis of bone marrow environment, *LepR*^*+*^ cells were clustered into four subpopulations.^203^ Cluster P1 (Mgp^high^) and P2 (Lpl^high^) highly expressed adipogenic markers, and were closely associated with sinusoidal capillaries. In contrast, cluster P3 (Wif1^high^) and P4 (Spp1^high^Ibsp^high^) were osteolineage-primed, and were located to the trabecular portion. Intriguingly, the P1 and P2 subsets were highly enriched for CFUs, and accounted for the majority of CFU-Fs of total LepR^+^ cells.^203^

In particular, a study combining scRNA-seq with spatial transcriptomics revealed a highly heterogenous landscape of the bone marrow stromal cells.^80^ In specific, CAR cells located at sinusoidal (Adipo-CAR, Cxcl12^+^Alpl^-^) and arteriolar/non-vascular (Osteo-CAR, Cxcl12^+^Alpl^+^) niches displayed distinct transcriptional profile favoring adipogenesis and osteogenesis respectively. Adipo-CAR cells exhibited a transcriptional pattern highly similar to LepR^+^ cells.^80^ However, previous work by Morrison laboratory showed LepR^+^ cells as main source for both bone and adipocytes in adult bone marrow, with the lepR^+^osteolectin^+^ subsets and *Adipoq-Cre*^*ER*^-expressing subsets giving rise to bone cells and adipocytes respectively.^64,128,133^ Thus, the relationship of LepR^+^, LepR^+^osteolectin^+^, Adipo-CAR and Osteo-CAR cells are to be elucidated.

Scadden Lab proposed taxonomy for bone marrow stroma that included 17 clusters.^204^ They consisted of MSCs (*Lepr*^+^, *Cxcl12*^+^), two osteo-lineage cell (OLC) subsets (*Bglap*^+^), four chondrocyte subsets (*Acan*^+^, *Col2a1*^+^), five fibroblast subsets (*S100a4*^+^), three bone marrow-derived endothelial cells (BMEC) subsets (*Cdh5*^+^), pericytes (*Acta2*^+^) and two clusters that uniformly expressed markers of chondrocytes, osteoblasts and fibroblasts. Here the Lepr^+^ MSCs highly expressed *lepr*, *adipoq*, *Cxcl12*, *Kitl* and *angiopoietin-1*, and were therefore annotated with pre-adipocytic features. Four subsets were further subclustered in Lepr-MSCs. Interestingly, one subset expressed a higher level of OLC-specific genes *Osx* and *Alpl*, indicating a continuous transition from Lepr-MSCs to OLCs. Besides, the two OLC clusters were originated from two distinct differentiation trajectories and exhibited different hematopoietic potential. The four chondrocyte subsets in the diffusion map, on the other hand, displayed a classical endochondral bone formation process.^204^

Besides the taxonomy of bone marrow stroma from transcriptional level, Scadden Lab further clustered the stroma subpopulations using mass cytometry (CyTOF)-based single-cell protein analysis. A total of 28 subsets were defined, among which 14 subsets expressed hematopoietic cytokines. Irradiation eradicated most of the populations, including LepR^+^ and Nes^+^ subsets, while CD73^+^ subset is found highly resilient to irradiation.^205^ Zhong et al. compared the bone marrow mesenchymal lineages from 3-month and 16-month-old *Col2-Cre;R26R-*tdTomato mice. A smaller number of early mesenchymal progenitors and a shift toward adipogenic transcriptome were observed in 16-months mice.^134^ Together, these studies demonstrate the extreme complexity of the bone marrow stromal components and provide valuable sources that could be exploited in the further work.

### Craniofacial bone at single-cell resolution

scRNA-seq is extensively applicated in craniofacial research.^143,206,207^ Calvaria cells collected from P4 pups were subjected to sequencing.^208^ Transcriptomes of the freshly isolated calvaria osteoblasts and cultured osteoblasts varied. Similar transcriptome was indicated between freshly isolated calvaria osteoblasts and long bone osteoblasts.^208^ Cells of coronal sutures from E15.5 and E17.5 were collected for scRNA-seq.^209^ Pre-osteoblasts between suture fronts and periosteum were distinct. A subpopulation above the cranial suture shared ligament characters and persisted into mice adulthood. A chondrogenic-like subpopulation was identified in the dura.^209^ Holmes et al. also isolated coronal suture cells of E16.5 and E18.5 for scRNA-seq.^210^ Seven populations were identified and the expression of *Hedgehog Interacting Protein* (*Hhip*) was found to mark mesenchymal population. *Hhip*-expressing cells at neonatal stage contributed to bone growth of calvaria.^210^ A single-cell atlas of human calvaria at 8 WPC was plotted. The data indicated that the proportions of osteoprogenitors and perichondrial mesenchymal stromal cells were much higher in calvarial bones compared to long bones. Twelve subsets were identified, of which the neural crest-derived cells (NCDC) cluster was PDGFRA^low^, PDPN^+^, and CADM1^+^, resided in the outer layer of the sagittal suture and mimicked the long bone eSSPC phenotype.^90^

## Disputes and challenges of SSCs

### Skeletal stem cells of different origins

A general agreement in SSC field is the diverse origins of SSCs. Unlike hematopoietic system that a group of clearly defined stem and progenitor cells generate the whole lineages arranged by different immunophenotypic markers, the skeletal elements are established and replenished with different sources.^117^ The human SSCs (hSSCs) and mouse SSCs (mSSCs) lineage trees plotted by Longaker Laboratory are currently the most comprehensive and plausible paradigm that can be referred in skeletal stem cell research.^38,66^ Nevertheless, some disputes regarding the mSSCs lineage map should be mentioned.

One is that in their experiments skeletal tissue was harvested from P3 mice when SOC and hematopoietic niches are not formed yet.^211^ One study detected the mSSCs in adult mice (8–16 weeks) and observed a contamination of *Col2.3*-GFP^+^ osteoblasts in endosteal mSSCs (~40% GFP^+^ cells in CD45^−^CD51^+^Thy^−^6C3^−^CD105^−^CD200^+^ cells) and progeny BCSP (~20% GFP^+^ cells in CD45^−^CD51^+^Thy^−^6C3^−^CD105^+^ cells) subpopulations, as well as in periosteal SSC and BCSP. Indeed, over 90% of *Col2.3*-GFP osteoblasts expressed CD200.^212^ Another study showed a rapid decrease of mSSCs and BCSP population with age. At E13.5, ~ 4 × 10^4^ SSCs were identified from 10^5^ cells, but the number plumped to less than 100 SSCs per 10^5^ cells at 8 weeks.^73^ Another issue is the location of mSSCs and BCSP, which were preferentially located at metaphysis and epiphysis. Few cells were detected in diaphysis and mSSCs failed to generate adipocytes even under strong external adipogenic induction in vitro.^73^ Therefore, mSSCs might produce a majority of bone components at embryonic and juvenile stage, but do not support hematopoiesis and bone remodeling at adult stage.

The perivascular CD45^−^CD31^−^Sca1^+^CD24^+^ progenitors in adult bone identified by Ambrosi et al. demonstrated another SSC source.^72^ This group of progenitors mainly resided in epiphysis and could be isolated from diaphysis as well. They exhibited a hematopoietic-supportive and adipogenic transcriptome, and were capable of generating bone, cartilage, stroma, and adipocytes when transplanted under renal capsule. scRNA-seq showed that mSSCs (CD45^−^CD51^+^Thy^−^6C3^−^CD105^−^CD200^+^ cells) and perivascular CD45^−^CD31^−^Sca1^+^CD24^+^ SSCs were separate clusters, supporting the notion of diverse origins of SSCs.^73^ Interestingly, a rare endothelial group of LNGFR^+^ cells in human fetal and regenerative bone marrow went through an endothelial-to-mesenchymal transition under the IL-33 signaling.^213^ Besides, Schwann cell precursors contributed to long bone development by transdifferentiating into mesenchymal cells.^141^ Neural crest-derived Nes^+^ cells differentiated into hematopoiesis-supporting SSCs as well.^214^

### Relation of SSCs labeled by reporter mouse models

There are around 20 types of transgene labels that are reported to identify SSCs and early progenitors. More than 30 types of Cre/Cre^ER^-driven reporter mice are constructed to characterize the spatiotemporal feature of SSCs. The information these studies provide is sometimes contradictory and partial overlap of different groups of SSCs are frequently observed. However, several important indications can be addressed from these overwhelming results.

One is the contribution of embryonic/early postnatal skeletal progenitors to postnatal SSCs. Current evidence supports that embryonic/early postnatal short-term progenitors in the perichondrium and growth plate invade into the bone marrow and re-establish into postnatal long-residing SSCs. For instance, cells marked by *Osx-Cre*^*ER*^ at perinatal stage generated long-lived Cxcl12^+^ stromal cells in the adult bone marrow.^69^ The same is true for most populations, such as *Col2-Cre*^*ER*^, *Sox9-Cre*^*ER*^, *Acan-Cre*^*ER*^, *Gli1-Cre*^*ER,*^ and *PTHrP-Cre*^*ER*^-marked cells perinatally.^41,67,98^ The de-differentiation of Col10a1^+^ hypertrophic chondrocytes into PDGFRα^H2B-GFP^-expressing SSCs were visualized.^122^ More importantly, by constructing a mutually exclusive tracing system, Zhou Laboratory unambiguously demonstrated that Col2^+^ progenitors gave rise to LepR^+^ SSCs.^77^

Second is the overlap of different populations marked by transgenes. At embryonic and perinatal stages, progenitors in the growth plate or immediately beneath it are simultaneously labeled by various transgenes. The postnatal marrow SSCs populations are also highly overlapped. Zhou et al. comprehensively characterized the CFU activity of various SSCs populations.^64^ These populations were differentially enriched for CFU-Fs, indicating some overlaps among them. Specifically, LepR^+^ cells denoted the PDGFRα^+^CD45^−^Ter119^−^ cells, uniformly expressed *Prx1*, and enriched for almost all the CFU-Fs. LepR^+^ cells also highly express *Scf*-GFP and *Cxcl12*-DsRed and *Nes*-GFP^low^, so it is thought that LepR^+^ cells denote CAR cells.

Third is that none of the transgenes faithfully label a homogenous SSC population. *Col2-Cre* labeled all the chondrocytes and progenies including osteoblasts, stromal cells and adipocytes.^67^ *PTHrP*-mCherry more specifically labeled SSCs and early progenitors, but their contribution to bone marrow was modest.^41^ Notably, Lepr expression was detected in osteo-lineage, endothelial cells, pericytes, and fibroblasts, besides in Lepr-SSCs.^204^ Therefore, the interpretation of phenotype by *Lepr-Cre*-driven gene alteration should be cautious. The employment of *LepR-Cre*^*ER*^ system might be a more reliable option than continuous *LepR-Cre* that is active throughout development. Alternatively, the dual-recombinase-activated lineage tracing (DeaLT) system that eliminates the interference from nontarget cells can delineate the stem cell fate more precisely.^54^

### Technique challenges in skeletal stem cell research

The mineralized structure makes skeletal research difficult. To isolate and culture SSCs, flushing of bone marrow, mechanical crush of the compact bone, enzymatically digestion, and cell sorting procedures are preferentially applied.^44,215,216^ Digestion releases the endosteal SSCs and yields a higher frequency of SSCs compared with flushing method. However, collagenase digestion is a stress that alters cellular transcriptome.^202,217^ It is also recommended to frequently change medium and adjust the trypsinization time to minimize hematopoietic contamination.^215^ Culture of the bone chips to allow fibroblast-like cells migration is also reported to yield stem cells.^216^ SSCs cultured as nonadherent mesenchymal spheres are reported as well.^96^ Low oxygen (2%) culture is also recommended.^38^ Nevertheless, different methods often yield different cell populations. Morphological difference can be easily discerned in cell culture. For example, the PDGFRα^+^Sca1^+^ cells were spindle-like while PDGFRα^+^Sca1^−^ cells appeared more rounded.^65^ Cell culture impacts SSCs properties as well. PDGFRα^+^Sca1^+^ cells lost the ability to home to bone marrow niches once cultured in vitro, while freshly isolated ones successfully reconstructed the vascular niche for hematopoiesis in lethally irradiated recipient mice.^65^

The functional assessment of SSCs after isolation is another topic. Tri-lineage differentiation assays in vitro are routinely harnessed with well-established protocols.^215,216^ The osteogenic and adipogenic differentiation was induced with monolayer cell culture, whereas chondrogenic differentiation was preferentially performed with cell pellet. In vivo assays were achieved by transplantation, in which SSCs were loaded into scaffold such as Matrigel and embedded under the skin, the mammary fat pad or kidney capsule of the immunodeficient mice.^38,41,44,71^ Weeks after transplantation the tissue was subject to histological analysis for the formation of bone, cartilage, stroma and adipose tissue. Bone marrow transplantation is an optional choice and is technically challenging because the mice have to survive irradiation first and then intravenous injection of SSCs is performed.^65,127,218^ Donor cells labeled with fluorescence and the reporter recipient mice are recommended as bone marrow comprises of huge amounts of cells difficult to discern. Real-time in vivo imaging is harnessed as well and SSCs are usually transplanted at calvarial site for visualization of cell migration and injury repair.^99^

The construction of Cre-loxP system for lineage tracing is challenging, as it requires both specific and efficient recombination. The mouse models constructed by *Nestin* transgenes are highly variable that *Nes-Cre* and *Nes*-GFP labeled different populations.^60,68^ *PDGFRα-Cre*^*ER*^, on the other hand, recombinased poorly in bone marrow PDGFRα^+^ cells, which were enriched for almost all the CFU-Fs.^61,64^

The sensitivity of single-cell sequencing should be rigorously interrogated as well. Members in Warman Laboratory treated mice with vehicle or sclerostin-neutralizing antibody, but failed to identify significant changes of bone anabolism-associated transcripts in osteoblasts.^208^ This study indicates the underpower of scRNA-seq at some settings. More confusingly, unexpected results are obtained from lineage tracing and scRNA-seq experiments even with robust tools. Unwanted cell types are unavoidably labeled or sorted, and hematopoietic lineages often contaminate the mesenchymal cells. Recent scRNA-seq results highlight the contamination issue.^123,134^ In a single-cell sequencing experiment performed by Ono group, the GFP^high^ population gated from *Cxcl12*^GFP/+^*;Cxcl12-cre*^*ER*^*;R26R*^tdTomato^ bone marrow were subjected to sequencing, but the results yield a substantial fraction (~26.2%) of *Cxcl12*-GFP^neg^ myeloid cells, lymphocytes, and erythroid cells.^123^ Similar contamination was observed in *Col2-Cre;R26R*-tdTomato mice. When the top 1% tdTomato^+^ endosteal cells were sorted for scRNA-seq, a large number of non-mesenchymal cells were observed. Surprisingly, these cells were validated for detectable *Tdt* and *Col2a1* expression.^134^ The reason and solution to such massive contamination remains to be answered.

The visualization of skeletal components remains an issue for decades.^219^ Long-term decalcification diminishes the epitopes on cells. Undecalcified frozen hard-tissue section using Kawamot’s Film method is now routinely harnessed in our lab for von-kossa staining and immunofluorescence.^220,221^ We also use the method developed by Adams Laboratory to prepare samples for three-dimension visualization.^222,223^ Tissue section with 20–100 μm thickness obtained with this method are subjected for immunostaining and confocal visualization. Besides, tissue-clearance technique is applied in our lab.^224,225^ For example, we are able to visualize the bone-implant interfaces after tissue-clearance with two-photon excitation microscope, which is almost unapplicable with other methods.

## Conclusions

This review summarizes the key findings of skeletal stem cells in the recent decade. The application of lineage tracing and high-throughput sequencing have greatly broadened and deepened our understanding of the skeletal development, homeostasis, and injury repair. Several aspects of skeletal stem cells are warranted for future exploration. New sample preparation methods and lineage tracing models that achieve efficient and reliable evaluation of the behavior of skeletal cells are to be explored. The crosstalk within bone marrow niches, and the mechanism that determines fate determination of SSCs remain to be elucidated. The cells and regulatory mechanism in craniofacial system exhibit some unique properties compared with long bones, and are to be uncovered as well.
